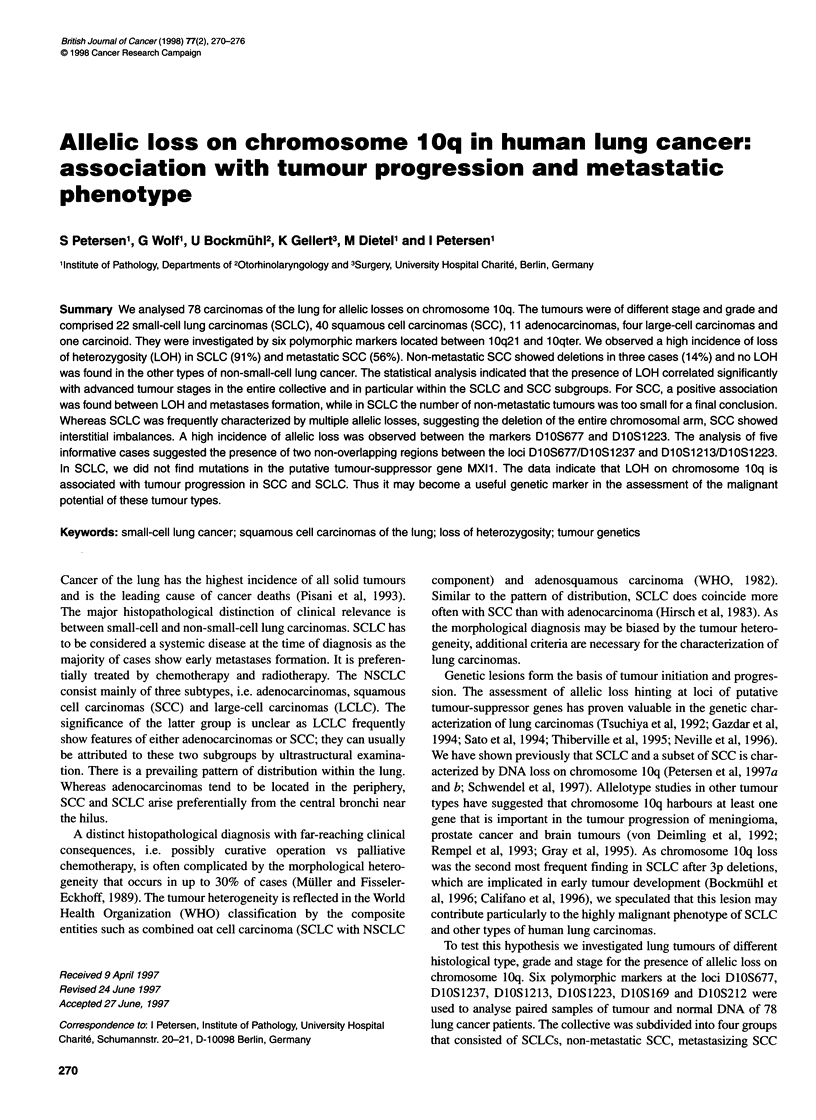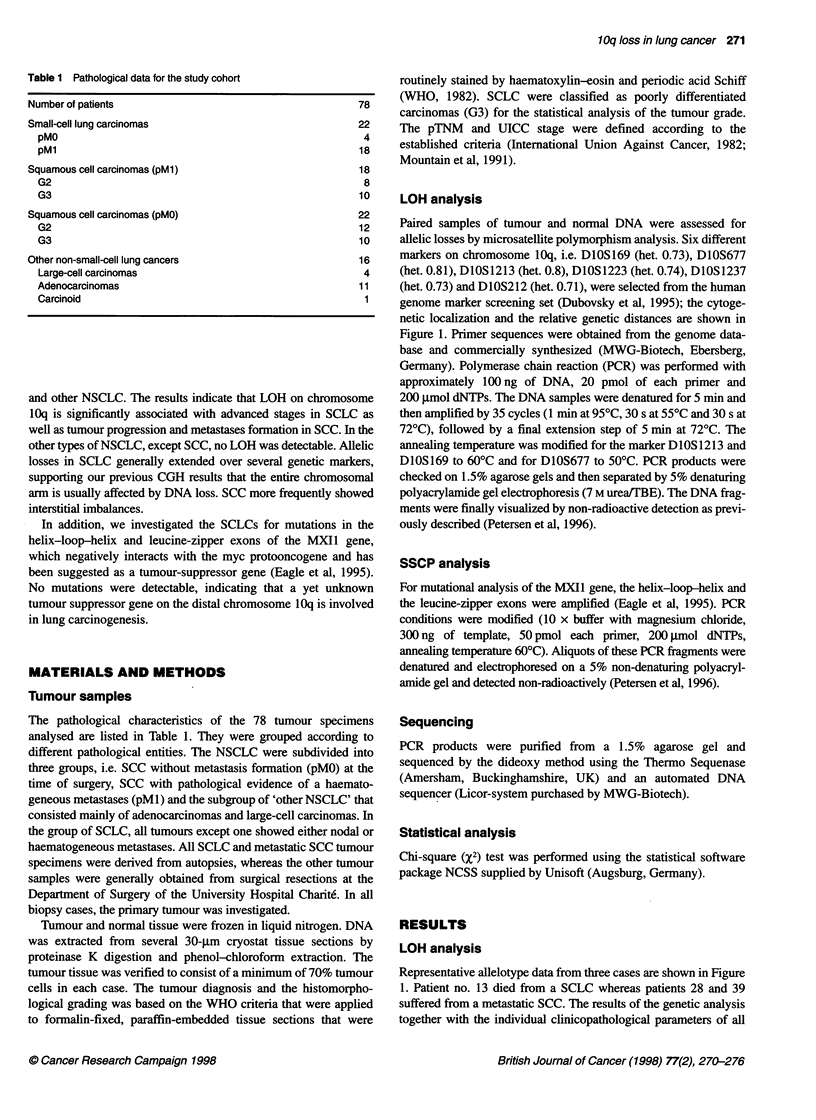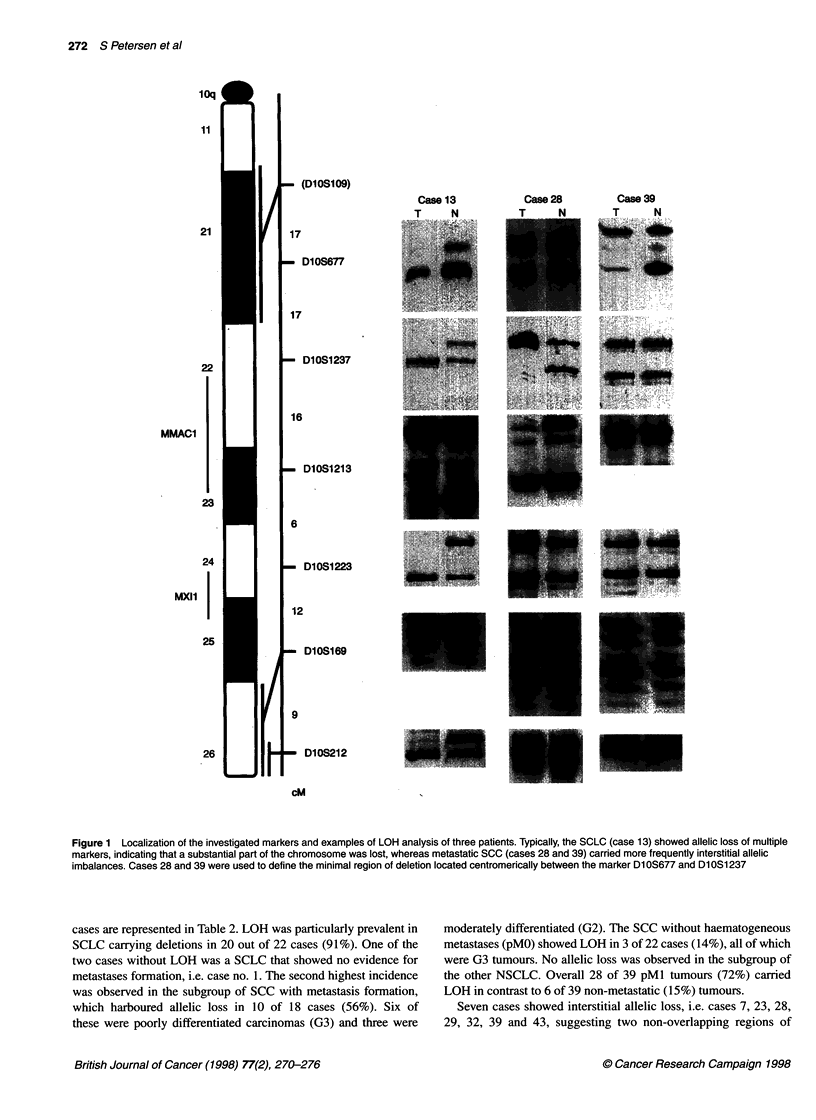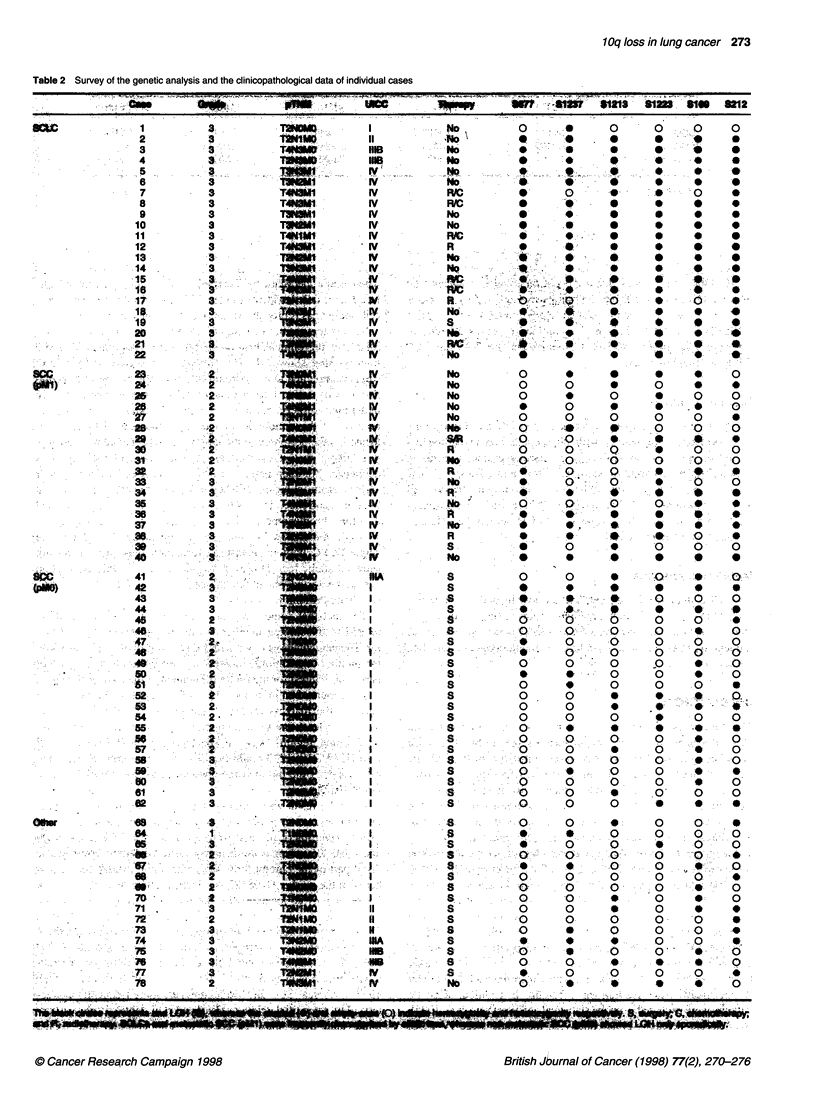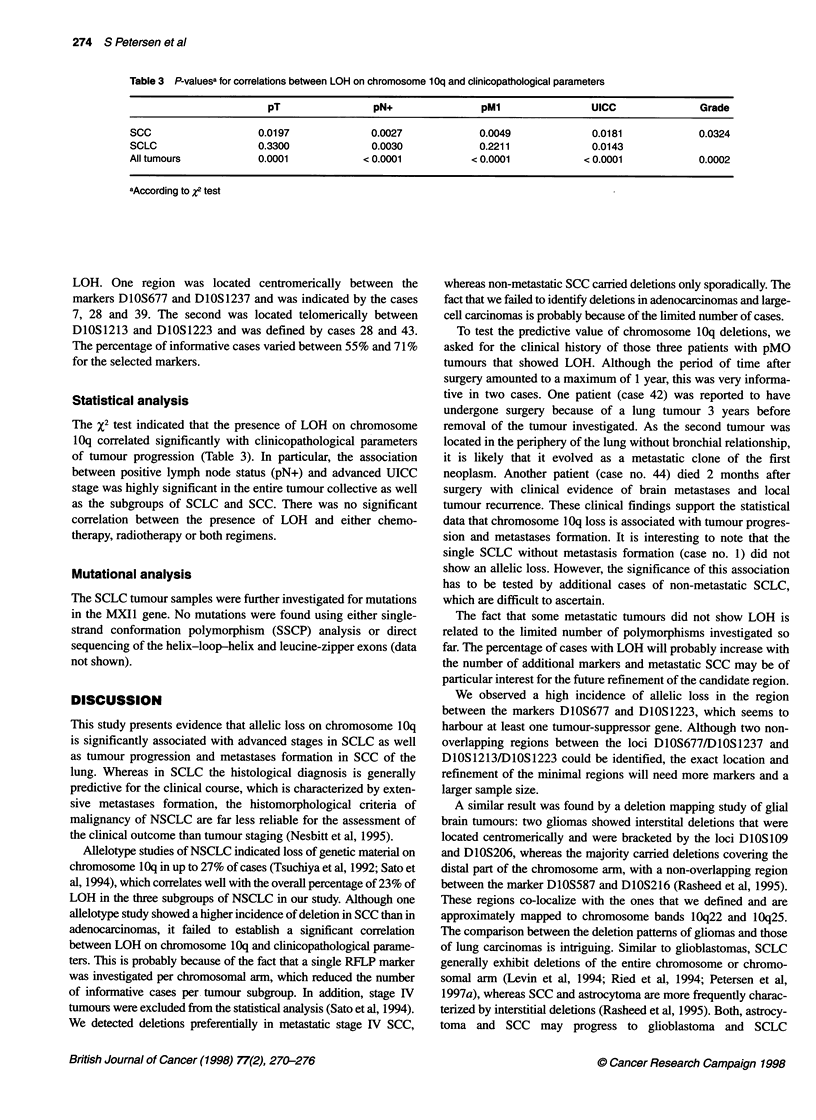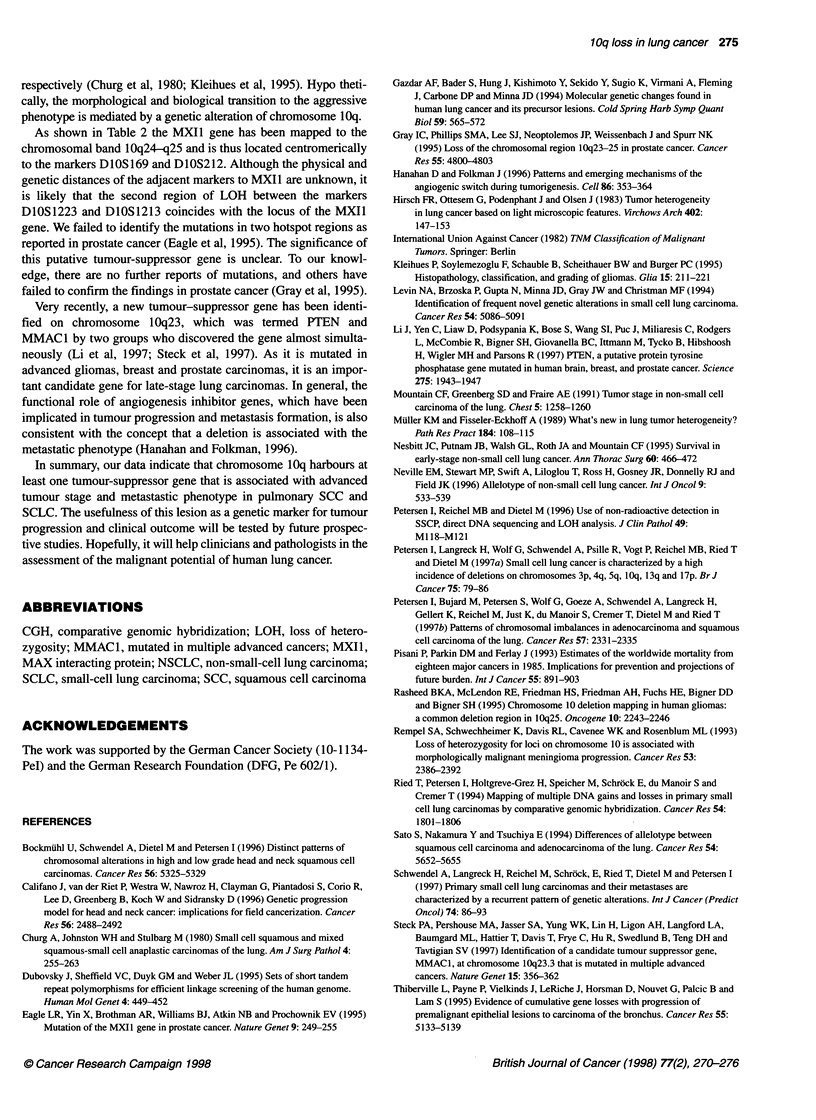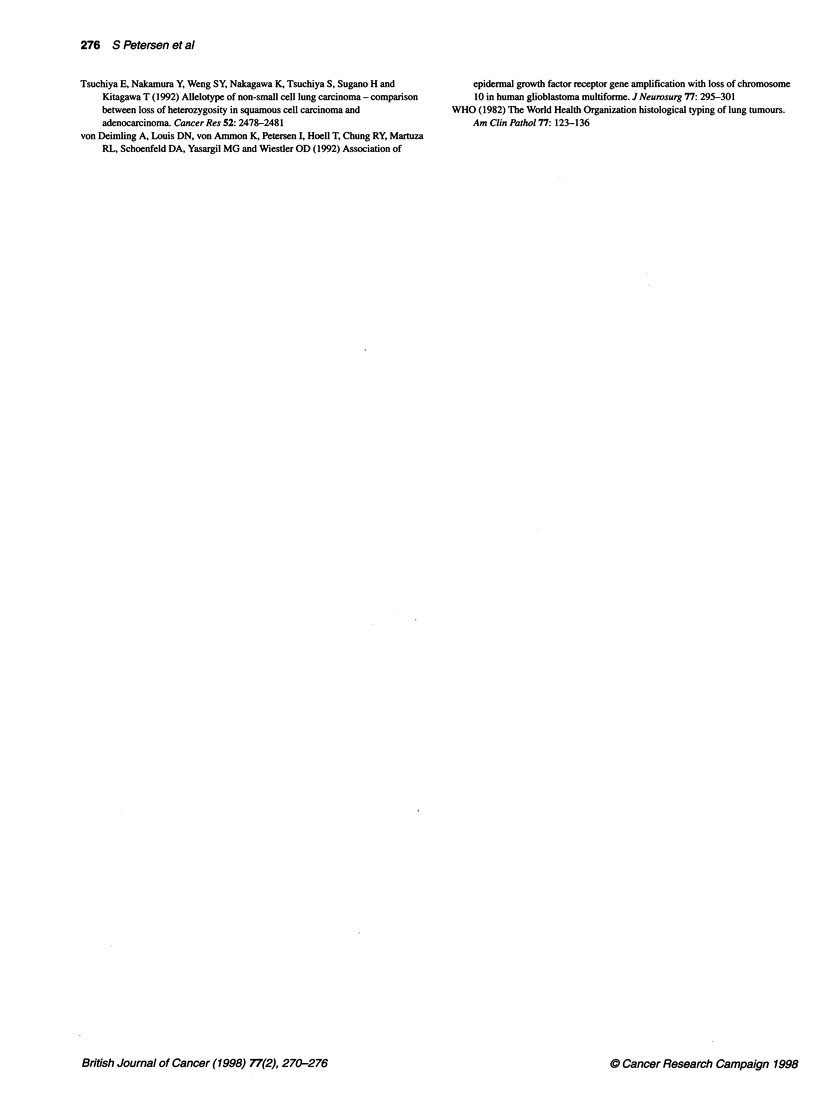# Allelic loss on chromosome 10q in human lung cancer: association with tumour progression and metastatic phenotype.

**DOI:** 10.1038/bjc.1998.43

**Published:** 1998

**Authors:** S. Petersen, G. Wolf, U. BockmÃ¼hl, K. Gellert, M. Dietel, I. Petersen

**Affiliations:** Institute of Pathology, Department of Otorhinolaryngology, University Hospital CharitÃ©, Berlin, Germany.

## Abstract

**Images:**


British Journal of Cancer (1998) 77(2), 270–276
© 1998 Cancer Research Campaign

### Allelic loss on chromosome 10q in human lung cancer: association with tumour progression and metastatic phenotype

S Petersen[1], G Wolf[1], U Bockmühl[2], K Gellert[3], M Dietel[1] and I Petersen[1]

[1]Institute of Pathology, Departments of [2]Otorhinolaryngology and [3]Surgery, University Hospital Charité, Berlin, Germany

**Summary** We analysed 78 carcinomas of the lung for allelic losses on chromosome 10q. The tumours were of different stage and grade and comprised 22 small-cell lung carcinomas (SCLC), 40 squamous cell carcinomas (SCC), 11 adenocarcinomas, four large-cell carcinomas and one carcinoid. They were investigated by six polymorphic markers located between 10q21 and 10qter. We observed a high incidence of loss of heterozygosity (LOH) in SCLC (91%) and metastatic SCC (56%). Non-metastatic SCC showed deletions in three cases (14%) and no LOH was found in the other types of non-small-cell lung cancer. The statistical analysis indicated that the presence of LOH correlated significantly with advanced tumour stages in the entire collective and in particular within the SCLC and SCC subgroups. For SCC, a positive association was found between LOH and metastases formation, while in SCLC the number of non-metastatic tumours was too small for a final conclusion. Whereas SCLC was frequently characterized by multiple allelic losses, suggesting the deletion of the entire chromosomal arm, SCC showed interstitial imbalances. A high incidence of allelic loss was observed between the markers D10S677 and D10S1223. The analysis of five informative cases suggested the presence of two non-overlapping regions between the loci D10S677/D10S1237 and D10S1213/D10S1223. In SCLC, we did not find mutations in the putative tumour-suppressor gene MXI1. The data indicate that LOH on chromosome 10q is associated with tumour progression in SCC and SCLC. Thus it may become a useful genetic marker in the assessment of the malignant potential of these tumour types.

**Keywords:** small-cell lung cancer; squamous cell carcinomas of the lung; loss of heterozygosity; tumour genetics

Cancer of the lung has the highest incidence of all solid tumours and is the leading cause of cancer deaths (Pisani et al, 1993). The major histopathological distinction of clinical relevance is between small-cell and non-small-cell lung carcinomas. SCLC has to be considered a systemic disease at the time of diagnosis as the majority of cases show early metastases formation. It is preferentially treated by chemotherapy and radiotherapy. The NSCLC consist mainly of three subtypes, i.e. adenocarcinomas, squamous cell carcinomas (SCC) and large-cell carcinomas (LCLC). The significance of the latter group is unclear as LCLC frequently show features of either adenocarcinomas or SCC; they can usually be attributed to these two subgroups by ultrastructural examination. There is a prevailing pattern of distribution within the lung. Whereas adenocarcinomas tend to be located in the periphery, SCC and SCLC arise preferentially from the central bronchi near the hilus.

A distinct histopathological diagnosis with far-reaching clinical consequences, i.e. possibly curative operation vs palliative chemotherapy, is often complicated by the morphological heterogeneity that occurs in up to 30% of cases (Müller and Fisseler-Eckhoff, 1989). The tumour heterogeneity is reflected in the World Health Organization (WHO) classification by the composite entities such as combined oat cell carcinoma (SCLC with NSCLC component) and adenosquamous carcinoma (WHO, 1982). Similar to the pattern of distribution, SCLC does coincide more often with SCC than with adenocarcinoma (Hirsch et al, 1983). As the morphological diagnosis may be biased by the tumour heterogeneity, additional criteria are necessary for the characterization of lung carcinomas.

Genetic lesions form the basis of tumour initiation and progression. The assessment of allelic loss hinting at loci of putative tumour-suppressor genes has proven valuable in the genetic characterization of lung carcinomas (Tsuchiya et al, 1992; Gazdar et al, 1994; Sato et al, 1994; Thiberville et al, 1995; Neville et al, 1996). We have shown previously that SCLC and a subset of SCC is characterized by DNA loss on chromosome 10q (Petersen et al, 1997*a* and *b*; Schwendel et al, 1997). Allelotype studies in other tumour types have suggested that chromosome 10q harbours at least one gene that is important in the tumour progression of meningioma, prostate cancer and brain tumours (von Deimling et al, 1992; Rempel et al, 1993; Gray et al, 1995). As chromosome 10q loss was the second most frequent finding in SCLC after 3p deletions, which are implicated in early tumour development (Bockmühl et al, 1996; Califano et al, 1996), we speculated that this lesion may contribute particularly to the highly malignant phenotype of SCLC and other types of human lung carcinomas.

To test this hypothesis we investigated lung tumours of different histological type, grade and stage for the presence of allelic loss on chromosome 10q. Six polymorphic markers at the loci D10S677, D10S1237, D10S1213, D10S1223, D10S169 and D10S212 were used to analyse paired samples of tumour and normal DNA of 78 lung cancer patients. The collective was subdivided into four groups that consisted of SCLCs, non-metastatic SCC, metastasizing SCC

*Received 9 April 1997*
*Revised 24 June 1997*
*Accepted 27 June, 1997*

*Correspondence to:* I Petersen, Institute of Pathology, University Hospital Charité, Schumannstr. 20–21, D-10098 Berlin, Germany

Table 1 Pathological data for the study cohort

| | |
|---|---|
| Number of patients | 78 |
| Small-cell lung carcinomas | 22 |
| pM0 | 4 |
| pM1 | 18 |
| Squamous cell carcinomas (pM1) | 18 |
| G2 | 8 |
| G3 | 10 |
| Squamous cell carcinomas (pM0) | 22 |
| G2 | 12 |
| G3 | 10 |
| Other non-small-cell lung cancers | 16 |
| Large-cell carcinomas | 4 |
| Adenocarcinomas | 11 |
| Carcinoid | 1 |

and other NSCLC. The results indicate that LOH on chromosome 10q is significantly associated with advanced stages in SCLC as well as tumour progression and metastases formation in SCC. In the other types of NSCLC, except SCC, no LOH was detectable. Allelic losses in SCLC generally extended over several genetic markers, supporting our previous CGH results that the entire chromosomal arm is usually affected by DNA loss. SCC more frequently showed interstitial imbalances.

In addition, we investigated the SCLCs for mutations in the helix–loop–helix and leucine-zipper exons of the MXI1 gene, which negatively interacts with the myc protooncogene and has been suggested as a tumour-suppressor gene (Eagle et al, 1995). No mutations were detectable, indicating that a yet unknown tumour suppressor gene on the distal chromosome 10q is involved in lung carcinogenesis.

### MATERIALS AND METHODS

### Tumour samples

The pathological characteristics of the 78 tumour specimens analysed are listed in Table 1. They were grouped according to different pathological entities. The NSCLC were subdivided into three groups, i.e. SCC without metastasis formation (pM0) at the time of surgery, SCC with pathological evidence of a haematogeneous metastases (pM1) and the subgroup of 'other NSCLC' that consisted mainly of adenocarcinomas and large-cell carcinomas. In the group of SCLC, all tumours except one showed either nodal or haematogeneous metastases. All SCLC and metastatic SCC tumour specimens were derived from autopsies, whereas the other tumour samples were generally obtained from surgical resections at the Department of Surgery of the University Hospital Charité. In all biopsy cases, the primary tumour was investigated.

Tumour and normal tissue were frozen in liquid nitrogen. DNA was extracted from several 30-μm cryostat tissue sections by proteinase K digestion and phenol–chloroform extraction. The tumour tissue was verified to consist of a minimum of 70% tumour cells in each case. The tumour diagnosis and the histomorphological grading was based on the WHO criteria that were applied to formalin-fixed, paraffin-embedded tissue sections that were routinely stained by haematoxylin–eosin and periodic acid Schiff (WHO, 1982). SCLC were classified as poorly differentiated carcinomas (G3) for the statistical analysis of the tumour grade. The pTNM and UICC stage were defined according to the established criteria (International Union Against Cancer, 1982; Mountain et al, 1991).

### LOH analysis

Paired samples of tumour and normal DNA were assessed for allelic losses by microsatellite polymorphism analysis. Six different markers on chromosome 10q, i.e. D10S169 (het. 0.73), D10S677 (het. 0.81), D10S1213 (het. 0.8), D10S1223 (het. 0.74), D10S1237 (het. 0.73) and D10S212 (het. 0.71), were selected from the human genome marker screening set (Dubovsky et al, 1995); the cytogenetic localization and the relative genetic distances are shown in Figure 1. Primer sequences were obtained from the genome database and commercially synthesized (MWG-Biotech, Ebersberg, Germany). Polymerase chain reaction (PCR) was performed with approximately 100 ng of DNA, 20 pmol of each primer and 200 μmol dNTPs. The DNA samples were denatured for 5 min and then amplified by 35 cycles (1 min at 95°C, 30 s at 55°C and 30 s at 72°C), followed by a final extension step of 5 min at 72°C. The annealing temperature was modified for the marker D10S1213 and D10S169 to 60°C and for D10S677 to 50°C. PCR products were checked on 1.5% agarose gels and then separated by 5% denaturing polyacrylamide gel electrophoresis (7 M urea/TBE). The DNA fragments were finally visualized by non-radioactive detection as previously described (Petersen et al, 1996).

### SSCP analysis

For mutational analysis of the MXI1 gene, the helix–loop–helix and the leucine-zipper exons were amplified (Eagle et al, 1995). PCR conditions were modified (10 × buffer with magnesium chloride, 300 ng of template, 50 pmol each primer, 200 μmol dNTPs, annealing temperature 60°C). Aliquots of these PCR fragments were denatured and electrophoresed on a 5% non-denaturing polyacrylamide gel and detected non-radioactively (Petersen et al, 1996).

### Sequencing

PCR products were purified from a 1.5% agarose gel and sequenced by the dideoxy method using the Thermo Sequenase (Amersham, Buckinghamshire, UK) and an automated DNA sequencer (Licor-system purchased by MWG-Biotech).

### Statistical analysis

Chi-square ($\chi^2$) test was performed using the statistical software package NCSS supplied by Unisoft (Augsburg, Germany).

### RESULTS

### LOH analysis

Representative allelotype data from three cases are shown in Figure 1. Patient no. 13 died from a SCLC whereas patients 28 and 39 suffered from a metastatic SCC. The results of the genetic analysis together with the individual clinicopathological parameters of all

© Cancer Research Campaign 1998

**Figure 1** Localization of the investigated markers and examples of LOH analysis of three patients. Typically, the SCLC (case 13) showed allelic loss of multiple markers, indicating that a substantial part of the chromosome was lost, whereas metastatic SCC (cases 28 and 39) carried more frequently interstitial allelic imbalances. Cases 28 and 39 were used to define the minimal region of deletion located centromerically between the marker D10S677 and D10S1237

cases are represented in Table 2. LOH was particularly prevalent in SCLC carrying deletions in 20 out of 22 cases (91%). One of the two cases without LOH was a SCLC that showed no evidence for metastases formation, i.e. case no. 1. The second highest incidence was observed in the subgroup of SCC with metastasis formation, which harboured allelic loss in 10 of 18 cases (56%). Six of these were poorly differentiated carcinomas (G3) and three were moderately differentiated (G2). The SCC without haematogeneous metastases (pM0) showed LOH in 3 of 22 cases (14%), all of which were G3 tumours. No allelic loss was observed in the subgroup of the other NSCLC. Overall 28 of 39 pM1 tumours (72%) carried LOH in contrast to 6 of 39 non-metastatic (15%) tumours.

Seven cases showed interstitial allelic loss, i.e. cases 7, 23, 28, 29, 32, 39 and 43, suggesting two non-overlapping regions of

© Cancer Research Campaign 1998

**Table 2** Survey of the genetic analysis and the clinicopathological data of individual cases

| | Case | Grade | pTNM | UICC | Therapy | S677 | S1237 | S1213 | S1223 | S169 | S212 |
|---|---|---|---|---|---|---|---|---|---|---|---|
| SCLC | 1 | 3 | T2N0M0 | I | No | ○ | ◐ | ○ | ○ | ○ | ○ |
| | 2 | 3 | T2N1M0 | II | No | ● | ● | ● | ● | ● | ◐ |
| | 3 | 3 | T4N3M0 | IIIB | No | ◐ | ◐ | ◐ | ● | ● | ● |
| | 4 | 3 | T2N3M0 | IIIB | No | ● | ◐ | ● | ◐ | ◐ | ◐ |
| | 5 | 3 | T3N3M1 | IV | No | ◐ | ● | ● | ◐ | ◐ | ● |
| | 6 | 3 | T3N2M1 | IV | No | ● | ● | ● | ◐ | ◐ | ● |
| | 7 | 3 | T4N3M1 | IV | R/C | ● | ○ | ◐ | ◐ | ○ | ◐ |
| | 8 | 3 | T4N3M1 | IV | R/C | ● | ● | ● | ◐ | ● | ◐ |
| | 9 | 3 | T3N3M1 | IV | No | ● | ◐ | ● | ◐ | ◐ | ◐ |
| | 10 | 3 | T3N2M1 | IV | No | ● | ◐ | ● | ◐ | ● | ◐ |
| | 11 | 3 | T4N1M1 | IV | R/C | ◐ | ◐ | ● | ◐ | ◐ | ● |
| | 12 | 3 | T4N3M1 | IV | R | ◐ | ● | ◐ | ◐ | ● | ● |
| | 13 | 3 | T2N2M1 | IV | No | ● | ● | ● | ● | ◐ | ● |
| | 14 | 3 | T3N3M1 | IV | No | ● | ● | ◐ | ◐ | ● | ● |
| | 15 | 3 | T4N3M1 | IV | R/C | ◐ | ● | ● | ● | ● | ● |
| | 16 | 3 | T4N3M1 | IV | R/C | ◐ | ◐ | ● | ◐ | ● | ◐ |
| | 17 | 3 | T3N1M1 | IV | R | ○ | ○ | ○ | ◐ | ○ | ◐ |
| | 18 | 3 | T4N3M1 | IV | No | ◐ | ● | ● | ◐ | ◐ | ● |
| | 19 | 3 | T3N3M1 | IV | S | ● | ● | ● | ◐ | ● | ● |
| | 20 | 3 | T3N3M1 | IV | No | ● | ◐ | ● | ● | ◐ | ● |
| | 21 | 3 | T3N2M1 | IV | R/C | ● | ◐ | ◐ | ● | ● | ● |
| | 22 | 3 | T4N3M1 | IV | No | ● | ◐ | ◐ | ● | ◐ | ● |
| SCC (pM1) | 23 | 2 | T3N0M1 | IV | No | ○ | ◐ | ● | ◐ | ◐ | ○ |
| | 24 | 2 | T4N0M1 | IV | No | ○ | ○ | ◐ | ○ | ◐ | ◐ |
| | 25 | 2 | T3N2M1 | IV | No | ○ | ◐ | ○ | ◐ | ○ | ○ |
| | 26 | 2 | T4N2M1 | IV | No | ◐ | ○ | ◐ | ◐ | ◐ | ○ |
| | 27 | 2 | T3N1M1 | IV | No | ○ | ○ | ○ | ○ | ○ | ◐ |
| | 28 | 2 | T3N0M1 | IV | No | ○ | ● | ● | ○ | ○ | ○ |
| | 29 | 2 | T4N3M1 | IV | S/R | ○ | ○ | ◐ | ● | ● | ◐ |
| | 30 | 2 | T2N1M1 | IV | R | ○ | ○ | ○ | ◐ | ○ | ○ |
| | 31 | 2 | T3N0M1 | IV | No | ○ | ○ | ○ | ○ | ○ | ○ |
| | 32 | 2 | T3N3M1 | IV | R | ◐ | ○ | ○ | ◐ | ● | ◐ |
| | 33 | 3 | T2N2M1 | IV | No | ◐ | ○ | ○ | ◐ | ○ | ○ |
| | 34 | 3 | T3N3M1 | IV | R | ◐ | ◐ | ● | ● | ● | ● |
| | 35 | 3 | T4N3M1 | IV | No | ○ | ○ | ○ | ○ | ◐ | ◐ |
| | 36 | 3 | T4N3M1 | IV | R | ◐ | ● | ● | ● | ● | ◐ |
| | 37 | 3 | T3N2M1 | IV | No | ◐ | ◐ | ◐ | ● | ◐ | ◐ |
| | 38 | 3 | T3N3M1 | IV | R | ◐ | ◐ | ● | ● | ○ | ◐ |
| | 39 | 3 | T2N3M1 | IV | S | ● | ○ | ◐ | ○ | ○ | ○ |
| | 40 | 3 | T4N3M1 | IV | No | ● | ◐ | ● | ● | ● | ● |
| SCC (pM0) | 41 | 2 | T2N2M0 | IIIA | S | ○ | ○ | ◐ | ○ | ◐ | ○ |
| | 42 | 3 | T2N0M0 | I | S | ● | ◐ | ● | ● | ◐ | ◐ |
| | 43 | 3 | T2N0M0 | I | S | ● | ◐ | ● | ○ | ○ | ○ |
| | 44 | 3 | T1N0M0 | I | S | ◐ | ◐ | ● | ● | ● | ● |
| | 45 | 2 | T2N0M0 | I | S | ○ | ○ | ○ | ○ | ○ | ◐ |
| | 46 | 3 | T2N0M0 | I | S | ○ | ○ | ○ | ○ | ◐ | ○ |
| | 47 | 2 | T1N0M0 | I | S | ◐ | ○ | ○ | ○ | ○ | ○ |
| | 48 | 2 | T2N0M0 | I | S | ◐ | ○ | ○ | ○ | ○ | ○ |
| | 49 | 2 | T2N0M0 | I | S | ○ | ○ | ○ | ○ | ◐ | ○ |
| | 50 | 2 | T2N0M0 | I | S | ◐ | ◐ | ○ | ○ | ◐ | ○ |
| | 51 | 3 | T2N0M0 | I | S | ○ | ◐ | ○ | ○ | ○ | ◐ |
| | 52 | 3 | T2N0M0 | I | S | ○ | ○ | ◐ | ◐ | ◐ | ○ |
| | 53 | 2 | T2N0M0 | I | S | ○ | ○ | ◐ | ◐ | ◐ | ◐ |
| | 54 | 2 | T2N0M0 | I | S | ○ | ○ | ○ | ◐ | ○ | ○ |
| | 55 | 2 | T2N0M0 | I | S | ○ | ◐ | ◐ | ◐ | ◐ | ◐ |
| | 56 | 2 | T2N0M0 | I | S | ○ | ○ | ○ | ○ | ◐ | ○ |
| | 57 | 2 | T2N0M0 | I | S | ○ | ○ | ◐ | ○ | ◐ | ○ |
| | 58 | 3 | T2N0M0 | I | S | ○ | ○ | ○ | ○ | ◐ | ○ |
| | 59 | 3 | T2N0M0 | I | S | ○ | ◐ | ○ | ○ | ◐ | ◐ |
| | 60 | 3 | T2N0M0 | I | S | ○ | ○ | ○ | ○ | ◐ | ○ |
| | 61 | 3 | T2N0M0 | I | S | ○ | ○ | ◐ | ○ | ○ | ○ |
| | 62 | 3 | T2N0M0 | I | S | ○ | ○ | ○ | ◐ | ◐ | ◐ |
| Other | 63 | 3 | T2N0M0 | I | S | ○ | ○ | ◐ | ○ | ○ | ◐ |
| | 64 | 1 | T1N0M0 | I | S | ◐ | ◐ | ○ | ○ | ○ | ○ |
| | 65 | 3 | T2N0M0 | I | S | ◐ | ○ | ○ | ◐ | ○ | ○ |
| | 66 | 2 | T2N0M0 | I | S | ○ | ○ | ○ | ○ | ○ | ◐ |
| | 67 | 2 | T1N0M0 | I | S | ◐ | ◐ | ○ | ○ | ◐ | ○ |
| | 68 | 2 | T1N0M0 | I | S | ○ | ○ | ○ | ○ | ○ | ◐ |
| | 69 | 2 | T2N0M0 | I | S | ○ | ○ | ○ | ○ | ◐ | ○ |
| | 70 | 2 | T1N0M0 | I | S | ○ | ○ | ◐ | ○ | ◐ | ○ |
| | 71 | 3 | T2N1M0 | II | S | ○ | ○ | ◐ | ○ | ◐ | ◐ |
| | 72 | 2 | T2N1M0 | II | S | ○ | ○ | ○ | ○ | ○ | ◐ |
| | 73 | 3 | T2N1M0 | II | S | ○ | ◐ | ○ | ○ | ○ | ◐ |
| | 74 | 3 | T3N2M0 | IIIA | S | ◐ | ◐ | ◐ | ○ | ○ | ◐ |
| | 75 | 3 | T4N2M0 | IIIB | S | ○ | ◐ | ○ | ○ | ◐ | ○ |
| | 76 | 3 | T4N3M1 | IIIB | S | ○ | ○ | ◐ | ◐ | ◐ | ○ |
| | 77 | 3 | T2N2M1 | IV | S | ◐ | ○ | ○ | ○ | ○ | ◐ |
| | 78 | 2 | T4N3M1 | IV | No | ○ | ◐ | ◐ | ◐ | ◐ | ○ |

The black circles represents and LOH (●), whereas the shaded (◐) and empty ones (○) indicate homozygosity and heterozygosity respectively. S, surgery; C, chemotherapy; and R, radiotherapy. SCLCs and metastatic SCC (pM1) were frequently characterized by allelic loss, whereas non-metastatic SCC (pM0) showed LOH only sporadically.

© Cancer Research Campaign 1998

**Table 3** *P*-values[a] for correlations between LOH on chromosome 10q and clinicopathological parameters

| | pT | pN+ | pM1 | UICC | Grade |
|---|---|---|---|---|---|
| SCC | 0.0197 | 0.0027 | 0.0049 | 0.0181 | 0.0324 |
| SCLC | 0.3300 | 0.0030 | 0.2211 | 0.0143 | |
| All tumours | 0.0001 | < 0.0001 | < 0.0001 | < 0.0001 | 0.0002 |

[a]According to $\chi^2$ test

LOH. One region was located centromerically between the markers D10S677 and D10S1237 and was indicated by the cases 7, 28 and 39. The second was located telomerically between D10S1213 and D10S1223 and was defined by cases 28 and 43. The percentage of informative cases varied between 55% and 71% for the selected markers.

### Statistical analysis

The $\chi^2$ test indicated that the presence of LOH on chromosome 10q correlated significantly with clinicopathological parameters of tumour progression (Table 3). In particular, the association between positive lymph node status (pN+) and advanced UICC stage was highly significant in the entire tumour collective as well as the subgroups of SCLC and SCC. There was no significant correlation between the presence of LOH and either chemotherapy, radiotherapy or both regimens.

### Mutational analysis

The SCLC tumour samples were further investigated for mutations in the MXI1 gene. No mutations were found using either single-strand conformation polymorphism (SSCP) analysis or direct sequencing of the helix–loop–helix and leucine-zipper exons (data not shown).

### DISCUSSION

This study presents evidence that allelic loss on chromosome 10q is significantly associated with advanced stages in SCLC as well as tumour progression and metastases formation in SCC of the lung. Whereas in SCLC the histological diagnosis is generally predictive for the clinical course, which is characterized by extensive metastases formation, the histomorphological criteria of malignancy of NSCLC are far less reliable for the assessment of the clinical outcome than tumour staging (Nesbitt et al, 1995).

Allelotype studies of NSCLC indicated loss of genetic material on chromosome 10q in up to 27% of cases (Tsuchiya et al, 1992; Sato et al, 1994), which correlates well with the overall percentage of 23% of LOH in the three subgroups of NSCLC in our study. Although one allelotype study showed a higher incidence of deletion in SCC than in adenocarcinomas, it failed to establish a significant correlation between LOH on chromosome 10q and clinicopathological parameters. This is probably because of the fact that a single RFLP marker was investigated per chromosomal arm, which reduced the number of informative cases per tumour subgroup. In addition, stage IV tumours were excluded from the statistical analysis (Sato et al, 1994). We detected deletions preferentially in metastatic stage IV SCC, whereas non-metastatic SCC carried deletions only sporadically. The fact that we failed to identify deletions in adenocarcinomas and large-cell carcinomas is probably because of the limited number of cases.

To test the predictive value of chromosome 10q deletions, we asked for the clinical history of those three patients with pMO tumours that showed LOH. Although the period of time after surgery amounted to a maximum of 1 year, this was very informative in two cases. One patient (case 42) was reported to have undergone surgery because of a lung tumour 3 years before removal of the tumour investigated. As the second tumour was located in the periphery of the lung without bronchial relationship, it is likely that it evolved as a metastatic clone of the first neoplasm. Another patient (case no. 44) died 2 months after surgery with clinical evidence of brain metastases and local tumour recurrence. These clinical findings support the statistical data that chromosome 10q loss is associated with tumour progression and metastases formation. It is interesting to note that the single SCLC without metastasis formation (case no. 1) did not show an allelic loss. However, the significance of this association has to be tested by additional cases of non-metastatic SCLC, which are difficult to ascertain.

The fact that some metastatic tumours did not show LOH is related to the limited number of polymorphisms investigated so far. The percentage of cases with LOH will probably increase with the number of additional markers and metastatic SCC may be of particular interest for the future refinement of the candidate region.

We observed a high incidence of allelic loss in the region between the markers D10S677 and D10S1223, which seems to harbour at least one tumour-suppressor gene. Although two non-overlapping regions between the loci D10S677/D10S1237 and D10S1213/D10S1223 could be identified, the exact location and refinement of the minimal regions will need more markers and a larger sample size.

A similar result was found by a deletion mapping study of glial brain tumours: two gliomas showed interstital deletions that were located centromerically and were bracketed by the loci D10S109 and D10S206, whereas the majority carried deletions covering the distal part of the chromosome arm, with a non-overlapping region between the marker D10S587 and D10S216 (Rasheed et al, 1995). These regions co-localize with the ones that we defined and are approximately mapped to chromosome bands 10q22 and 10q25. The comparison between the deletion patterns of gliomas and those of lung carcinomas is intriguing. Similar to glioblastomas, SCLC generally exhibit deletions of the entire chromosome or chromosomal arm (Levin et al, 1994; Ried et al, 1994; Petersen et al, 1997*a*), whereas SCC and astrocytoma are more frequently characterized by interstitial deletions (Rasheed et al, 1995). Both, astrocytoma and SCC may progress to glioblastoma and SCLC

© Cancer Research Campaign 1998

respectively (Churg et al, 1980; Kleihues et al, 1995). Hypo thetically, the morphological and biological transition to the aggressive phenotype is mediated by a genetic alteration of chromosome 10q.

As shown in Table 2 the MXI1 gene has been mapped to the chromosomal band 10q24–q25 and is thus located centromerically to the markers D10S169 and D10S212. Although the physical and genetic distances of the adjacent markers to MXI1 are unknown, it is likely that the second region of LOH between the markers D10S1223 and D10S1213 coincides with the locus of the MXI1 gene. We failed to identify the mutations in two hotspot regions as reported in prostate cancer (Eagle et al, 1995). The significance of this putative tumour-suppressor gene is unclear. To our knowledge, there are no further reports of mutations, and others have failed to confirm the findings in prostate cancer (Gray et al, 1995).

Very recently, a new tumour-suppressor gene has been identified on chromosome 10q23, which was termed PTEN and MMAC1 by two groups who discovered the gene almost simultaneously (Li et al, 1997; Steck et al, 1997). As it is mutated in advanced gliomas, breast and prostate carcinomas, it is an important candidate gene for late-stage lung carcinomas. In general, the functional role of angiogenesis inhibitor genes, which have been implicated in tumour progression and metastasis formation, is also consistent with the concept that a deletion is associated with the metastatic phenotype (Hanahan and Folkman, 1996).

In summary, our data indicate that chromosome 10q harbours at least one tumour-suppressor gene that is associated with advanced tumour stage and metastastic phenotype in pulmonary SCC and SCLC. The usefulness of this lesion as a genetic marker for tumour progression and clinical outcome will be tested by future prospective studies. Hopefully, it will help clinicians and pathologists in the assessment of the malignant potential of human lung cancer.

### ABBREVIATIONS

CGH, comparative genomic hybridization; LOH, loss of heterozygosity; MMAC1, mutated in multiple advanced cancers; MXI1, MAX interacting protein; NSCLC, non-small-cell lung carcinoma; SCLC, small-cell lung carcinoma; SCC, squamous cell carcinoma

### ACKNOWLEDGEMENTS

The work was supported by the German Cancer Society (10-1134-PeI) and the German Research Foundation (DFG, Pe 602/1).

### REFERENCES

Bockmühl U, Schwendel A, Dietel M and Petersen I (1996) Distinct patterns of chromosomal alterations in high and low grade head and neck squamous cell carcinomas. *Cancer Res* **56**: 5325–5329

Califano J, van der Riet P, Westra W, Nawroz H, Clayman G, Piantadosi S, Corio R, Lee D, Greenberg B, Koch W and Sidransky D (1996) Genetic progression model for head and neck cancer: implications for field cancerization. *Cancer Res* **56**: 2488–2492

Churg A, Johnston WH and Stulbarg M (1980) Small cell squamous and mixed squamous-small cell anaplastic carcinomas of the lung. *Am J Surg Pathol* **4**: 255–263

Dubovsky J, Sheffield VC, Duyk GM and Weber JL (1995) Sets of short tandem repeat polymorphisms for efficient linkage screening of the human genome. *Human Mol Genet* **4**: 449–452

Eagle LR, Yin X, Brothman AR, Williams BJ, Atkin NB and Prochownik EV (1995) Mutation of the MXI1 gene in prostate cancer. *Nature Genet* **9**: 249–255

Gazdar AF, Bader S, Hung J, Kishimoto Y, Sekido Y, Sugio K, Virmani A, Fleming J, Carbone DP and Minna JD (1994) Molecular genetic changes found in human lung cancer and its precursor lesions. *Cold Spring Harb Symp Quant Biol* **59**: 565–572

Gray IC, Phillips SMA, Lee SJ, Neoptolemos JP, Weissenbach J and Spurr NK (1995) Loss of the chromosomal region 10q23–25 in prostate cancer. *Cancer Res* **55**: 4800–4803

Hanahan D and Folkman J (1996) Patterns and emerging mechanisms of the angiogenic switch during tumorigenesis. *Cell* **86**: 353–364

Hirsch FR, Ottesem G, Podenphant J and Olsen J (1983) Tumor heterogeneity in lung cancer based on light microscopic features. *Virchows Arch* **402**: 147–153

International Union Against Cancer (1982) *TNM Classification of Malignant Tumors*. Springer: Berlin

Kleihues P, Soylemezoglu F, Schauble B, Scheithauer BW and Burger PC (1995) Histopathology, classification, and grading of gliomas. *Glia* **15**: 211–221

Levin NA, Brzoska P, Gupta N, Minna JD, Gray JW and Christman MF (1994) Identification of frequent novel genetic alterations in small cell lung carcinoma. *Cancer Res* **54**: 5086–5091

Li J, Yen C, Liaw D, Podsypania K, Bose S, Wang SI, Puc J, Miliaresis C, Rodgers L, McCombie R, Bigner SH, Giovanella BC, Ittmann M, Tycko B, Hibshoosh H, Wigler MH and Parsons R (1997) PTEN, a putative protein tyrosine phosphatase gene mutated in human brain, breast, and prostate cancer. *Science* **275**: 1943–1947

Mountain CF, Greenberg SD and Fraire AE (1991) Tumor stage in non-small cell carcinoma of the lung. *Chest* **5**: 1258–1260

Müller KM and Fisseler-Eckhoff A (1989) What's new in lung tumor heterogeneity? *Path Res Pract* **184**: 108–115

Nesbitt JC, Putnam JB, Walsh GL, Roth JA and Mountain CF (1995) Survival in early-stage non-small cell lung cancer. *Ann Thorac Surg* **60**: 466–472

Neville EM, Stewart MP, Swift A, Liloglou T, Ross H, Gosney JR, Donnelly RJ and Field JK (1996) Allelotype of non-small cell lung cancer. *Int J Oncol* **9**: 533–539

Petersen I, Reichel MB and Dietel M (1996) Use of non-radioactive detection in SSCP, direct DNA sequencing and LOH analysis. *J Clin Pathol* **49**: M118–M121

Petersen I, Langreck H, Wolf G, Schwendel A, Psille R, Vogt P, Reichel MB, Ried T and Dietel M (1997*a*) Small cell lung cancer is characterized by a high incidence of deletions on chromosomes 3p, 4q, 5q, 10q, 13q and 17p. *Br J Cancer* **75**: 79–86

Petersen I, Bujard M, Petersen S, Wolf G, Goeze A, Schwendel A, Langreck H, Gellert K, Reichel M, Just K, du Manoir S, Cremer T, Dietel M and Ried T (1997*b*) Patterns of chromosomal imbalances in adenocarcinoma and squamous cell carcinoma of the lung. *Cancer Res* **57**: 2331–2335

Pisani P, Parkin DM and Ferlay J (1993) Estimates of the worldwide mortality from eighteen major cancers in 1985. Implications for prevention and projections of future burden. *Int J Cancer* **55**: 891–903

Rasheed BKA, McLendon RE, Friedman HS, Friedman AH, Fuchs HE, Bigner DD and Bigner SH (1995) Chromosome 10 deletion mapping in human gliomas: a common deletion region in 10q25. *Oncogene* **10**: 2243–2246

Rempel SA, Schwechheimer K, Davis RL, Cavenee WK and Rosenblum ML (1993) Loss of heterozygosity for loci on chromosome 10 is associated with morphologically malignant meningioma progression. *Cancer Res* **53**: 2386–2392

Ried T, Petersen I, Holtgreve-Grez H, Speicher M, Schröck E, du Manoir S and Cremer T (1994) Mapping of multiple DNA gains and losses in primary small cell lung carcinomas by comparative genomic hybridization. *Cancer Res* **54**: 1801–1806

Sato S, Nakamura Y and Tsuchiya E (1994) Differences of allelotype between squamous cell carcinoma and adenocarcinoma of the lung. *Cancer Res* **54**: 5652–5655

Schwendel A, Langreck H, Reichel M, Schröck, E, Ried T, Dietel M and Petersen I (1997) Primary small cell lung carcinomas and their metastases are characterized by a recurrent pattern of genetic alterations. *Int J Cancer (Predict Oncol)* **74**: 86–93

Steck PA, Pershouse MA, Jasser SA, Yung WK, Lin H, Ligon AH, Langford LA, Baumgard ML, Hattier T, Davis T, Frye C, Hu R, Swedlund B, Teng DH and Tavtigian SV (1997) Identification of a candidate tumour suppressor gene, MMAC1, at chromosome 10q23.3 that is mutated in multiple advanced cancers. *Nature Genet* **15**: 356–362

Thiberville L, Payne P, Vielkinds J, LeRiche J, Horsman D, Nouvet G, Palcic B and Lam S (1995) Evidence of cumulative gene losses with progression of premalignant epithelial lesions to carcinoma of the bronchus. *Cancer Res* **55**: 5133–5139

© Cancer Research Campaign 1998

Tsuchiya E, Nakamura Y, Weng SY, Nakagawa K, Tsuchiya S, Sugano H and Kitagawa T (1992) Allelotype of non-small cell lung carcinoma – comparison between loss of heterozygosity in squamous cell carcinoma and adenocarcinoma. *Cancer Res* **52**: 2478–2481

von Deimling A, Louis DN, von Ammon K, Petersen I, Hoell T, Chung RY, Martuza RL, Schoenfeld DA, Yasargil MG and Wiestler OD (1992) Association of epidermal growth factor receptor gene amplification with loss of chromosome 10 in human glioblastoma multiforme. *J Neurosurg* **77**: 295–301

WHO (1982) The World Health Organization histological typing of lung tumours. *Am Clin Pathol* **77**: 123–136

© Cancer Research Campaign 1998